# Severe Rhabdomyolysis Associated in Meningococcemia

**DOI:** 10.4103/0974-777X.68546

**Published:** 2010

**Authors:** Sharma Vishal, Agarwal P Mukul, Subhash Giri

**Affiliations:** *Department of Medicine, University College of Medical Sciences and GTB Hospital, Delhi, India*

Sir,

We read with interest the article on rhabdomyolysis secondary to staphylococcal endocarditis.[1] We would like to share our experience with a case of severe rhabdomyolysis secondary to meningococcemia.

**Figure 1:**
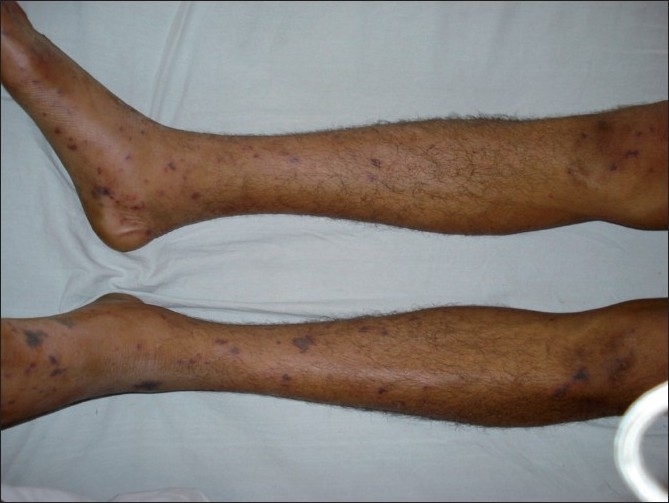
Photograph showing purpuric rash of meningococcemia

A 22-year-old male, labourer by occupation, presented with a 2-day history of fever. The fever was abrupt in onset and was associated with chills and rigors. The patient had also developed a purpuric rash and altered sensorium for 1 day. There was no history of any seizures. At the time of presentation, the patient was unconscious and in hypotension (blood pressure of 70/48 mmHg). He had tachycardia (pulse 118/min, regular) and was tachypneic (RR 28/min). He had a purpuric rash over his extremities [Figure 1]. He also had neck rigidity, but moved all limbs to pain, and his plantars were flexor. Fundus revealed no papilledema. In view of the presentation, a working diagnosis of meningococcemia with meningitis was made and the patient was managed with volume resuscitation, intravenous ceftriaxone and hydrocortisone. His blood pressure improved initially. Noncontrast computed tomography (CT) of the head was unremarkable. A lumbar puncture was performed and the cerebrospinal fluid revealed protein of 112 mg/dL, sugar of 12 mg/ dL, cells 2,300 with 95% polymorphs and gram stain showing gram-negative diplococcici. Latex agglutination test was positive for Group A meningococcal infection.

Over his stay, his urine output was low (100 mL over 12 h) and urine dipstick showed presence of blood. His renal functions were deranged (serum creatinine 4.3 ng/ mL, blood urea 242 mg/dL, serum Na+ 134, serum K 5.5 meq/L). Serum creatine kinase was 32,220 U/L. Other investigations revealed Hb 11.8 gm%, TLC 23,000, platelet count 48,000/µL, serum bilirubin 1.9, SGPT 96 U/L. Urine myoglobin was not performed. Although the patient was planned for hemodialysis, he developed hypotension and, in spite of all measures, including vasopressor support, the patient died.

The above case highlights the presence of severe rhabdomyolysis in case of bacterial septicaemia secondary to meningococcal infection. Although rhabdomyolysis has been reported with meningococcal infection, we are not aware of any report of renal failure secondary to rhabdomyolysis with meningococcemia.[2,3] Rhabdomyolysis can therefore be another cause of acute renal shutdown in sepsis apart from septic acute tubular necrosis. Clinicians should be aware of this possibility and appropriate management, including renal replacement, should be initiated when indicated.
